# Designing Meaningful Engagement for Older Adults: An Evaluation of Participation in a Five‐Day Co‐Design Sprint

**DOI:** 10.1111/hex.70555

**Published:** 2026-01-12

**Authors:** Susanna Nordin, Aaron Davis, France Légaré, Allyson Jones, Louise Meijering, Marie Elf

**Affiliations:** ^1^ School of Health and Welfare Dalarna University Falun Sweden; ^2^ UniSA Creative University of South Australia Adelaide South Australia Australia; ^3^ Department of Family Medicine and Emergency Medicine Université Laval Quebec City Québec Canada; ^4^ Department of Physical Therapy University of Alberta Edmonton Alberta Canada; ^5^ Faculty of Spatial Sciences University of Groningen Groningen the Netherlands

**Keywords:** co‐design, digital technology, evaluation, facilitation, participation, value‐sensitive design, welldoing

## Abstract

**Background:**

Co‐design is increasingly used to involve older adults in the development of digital tools, yet less attention has been given to how to engage them effectively in these processes. Older adults represent a highly diverse population, but co‐design practices often overlook this heterogeneity and rely on limited representation. Similarly, co‐design approaches vary widely in structure and degree of user involvement. While evaluations often focus on outcomes such as usability and technical performance, fewer studies explore the value of participation itself.

**Aim:**

This study evaluates the process of a 5‐day online design sprint where participants, including older adults, healthcare and technology professionals, researchers, facilitators and business representatives, collaborated to develop housing‐related digital support tools.

**Methods:**

A hybrid thematic analysis process links process documentation and participant reflections with value‐based frameworks to examine how different elements of the process created or inhibited value for participants.

**Results:**

The findings highlight how the structure of the design sprint, together with specific facilitation practices, fostered respectful dialogue, trust, autonomy and a sense of ownership among participants. The evaluation describes how the process satisfied participants' collectively prioritised values, and their individual needs.

**Conclusions:**

The study highlights that co‐design is not a neutral method, but a practice with social, ethical and emotional consequences. Effective facilitation is essential to support agency, ensure psychological safety and promote authentic collaboration. Recognising the heterogeneity of older adults and aligning facilitation strategies with participants' values and needs can enhance both the relevance of digital innovations and the meaningfulness of the co‐design experience itself.

**Patient and Public Contribution:**

The project was supported by a reference group that was actively involved throughout. The group included, for instance, representatives from the municipality, regional authorities and a pensioners' organisation.

## 1 Introduction

Participation in the co‐design of systems and services is well established as being associated with better project outcomes, yet how we should co‐design remains the subject of debate in the literature [1]. The meaningful involvement of people in the design of products, services and systems is often described as though there is a singular or correct formula for doing so. In reality, however, there are a plethora of ways of designing and facilitating co‐design processes [2]. In this paper, we evaluate a co‐design process that was undertaken with older adults to create a digital support tool for making decisions about housing futures, with a primary focus on the process itself. We explore the specific experience of participants, drawing insights into how different approaches and processes may be linked with outcomes.

## 2 Background

Older adults are increasingly expected to use digital services in everyday life [3]; nevertheless, they are rarely meaningfully involved in the design of these technologies [4]. At the same time, many faces difficult decisions about where and how to live as their housing needs change [5]. Their housing decisions often emerge from complex life transitions such as declining health, bereavement or increased care needs. Housing options may include adapting the current home, relocating to more accessible housing or considering supported living options [6, 7]. Digital tools have been proposed to support older adults in making informed value‐congruent housing decisions by enabling reflection, access to relevant information and shared decision‐making. However, these tools often fall short due to a lack of meaningful involvement of the intended users in their development.

Various co‐design approaches have become increasingly common for developing digital solutions and services in engaging different stakeholders. However, there is still limited knowledge about how and in what way the co‐design process generates value for all participants involved, and in particular for older people. Co‐designing tools in partnership with older adults offers an opportunity to involving users meaningfully in the development of these digital products. Co‐design methods can vary in the extent of user involvement, from occasional consultation to continuous collaboration and shared decision‐making [8, 9]. The approach is particularly relevant for ageing‐related technologies, as it offers opportunities to engage older adults in shaping solutions that affect their lives. However, older adults are a highly heterogeneous group, differing in age, health, cognitive ability, cultural background and socioeconomic status [10, 11, 12]. This diversity is frequently overlooked in co‐design practices, which often assume uniformity in participants' needs and digital competencies, operating under the assumption that a small group can represent the needs of all older adults.

At the same time, there is also heterogeneity in the types of co‐design processes and approaches that can be used to engage older adults.

There are many different approaches to evaluating co‐design, but much of the literature has in the past focused on final product outcomes, usability, satisfaction and technical performance rather than on the value of participation itself [13]. Recent developments in process evaluation, such as the PROcesS Evaluation framework for CO‐creation (PROSECO) framework [14], have foregrounded participation as an important part of evaluation. In the present study, we apply two complementary frameworks, each taking a subtly different approach to the concept of describing values and value: Value‐sensitive design (VSD) [15, 16] and Welldoing [17].

### 2.1 Theoretical Framework

VSD is a theory‐based approach to technology design that incorporates human values in a principled and thorough way during the design process [16]. Values can be described as ideals or interests among users and establish a person‐centred definition of success [18]. Originally applied to technology development [19, 20], VSD has expanded to healthcare contexts including mental healthcare [21], cardiovascular care [22] and dementia care [23, 24].

Well‐doing is a theory‐based approach to the design of co‐design processes [17]. This framework uses Max‐Neef's needs and satisfiers theory as a basis for planning and evaluating participatory engagement in a way that can maximise intrinsic value through the satisfaction of human needs [25]. Some processes or experiences during co‐design can genuinely support well‐being, while others may have a detrimental impact. In co‐design practices, it is possible for processes to meet one need while simultaneously preventing other needs from being satisfied (pseudo‐satisfier). For instance, overly authoritarian facilitation can build understanding of the topic among participants but restrict opportunities for participation, creation, identify formation or freedom [25]. Other processes satisfy only one level of need, such as the need for participation, but without providing an opportunity for creativity in contributions (singular satisfier). The most meaningful experiences, synergic satisfiers, enable the satisfaction of multiple needs and are therefore more likely to enhance well‐being [17].

There is a lack of insight into how older adults experience co‐design emotionally, socially, culturally and ethically and whether participation in these processes satisfies deeper human needs such as autonomy, identity or belonging. These gaps raise critical questions, such as what values emerge in the co‐design process? What needs are fulfilled or left unmet? And how can participation itself be evaluated as a meaningful outcome? This study addresses this gap in the context of older adults by exploring the co‐design process not just as a means to develop usable products, but as a context in which value is created, negotiated and experienced.

The present study aims to generate knowledge on how value emerges through co‐design with diverse stakeholders, including older adults, in the context of digital housing solutions. Specifically, we explore how values articulated during a co‐design process can inform both the design outcomes and the evaluation of participant engagement. In doing so, we aim to advance understanding of inclusive and ethically grounded co‐design practices in the fields of ageing and technology development.

## 3 Methods

### 3.1 Study Design

This study builds on previous similar work (such as [26]), using a case study approach [27] to explore the experience of participants in a co‐design process, and to link these experiences with co‐design strategies and processes that were used. The study evaluates the case of a 5‐day design sprint [28] and is part of the COORDINATEs project [29], which aimed to develop digital solutions to support housing decisions for older adults. Data describing the case includes a range of sources, including observations, participant‐generated materials and semi‐structured interviews to build a rich understanding of older adults' experiences in the case study project.

### 3.2 Patient and Public Involvement

From the outset, the project was supported by a reference group consisting of representatives from the municipality, regional authorities, a pensioners' organisation and academia. The group included managers responsible for care services for older adults, home care staff, senior advisors, individuals responsible for assistive technologies, board members of a pensioners' organisation and researchers with expertise in care environments and technology. The reference group played an important role in strengthening the project by providing input, feedback and advice. Regular meetings were held several times per year. In collaboration with the project team, the reference group contributed, for example, to the development of a semi‐structured interview guide informed by the overarching research questions.

### 3.3 Data Collection

Data were collected before, during and after the co‐design sprint. Before the sprint, processes and facilitation approaches were developed and documented. During the sprint, the co‐design team made observations and took field notes, which were documented and collated at the end of each day. Core aspects and impressions from the group's work were also discussed informally in unstructured daily reflections. After the sprint, individual interviews were conducted via telephone within 2 weeks to capture stakeholders' values related to the co‐design process. A semi‐structured guide with open‐ended questions was used to support the conversations. Areas of inquiry were centred around key human values from the process, such as respect, inclusion and collaboration. For example, participants were asked about their motivation for taking part in the design sprint, their experiences of collaboration and group dynamics, and the opportunities for different perspectives and values to be expressed. During the interviews, follow‐up questions were asked to clarify thoughts and meanings. The interviews lasted between 19 and 53 min and were audio‐recorded and transcribed verbatim. The semi‐structured interview guide can be found in Supporting Information S1.

Participant quotations are identified using unique participant codes (e.g., IP1) to enable distinction between individual contributions among the 18 interviewed participants while maintaining confidentiality.

### 3.4 Data Analysis

The data were analysed using a hybrid thematic analysis approach [30]. This enables participants' experiences to be explored through multiple theoretical lenses, both inductively and deductively. The data were first analysed using an inductive thematic analysis process [31] to identify recurring patterns in how participants described their involvement in the co‐design process. Transcripts were read in full, coded for meaning, and themes were developed iteratively through comparison, refinement and re‐engagement with the data by two of the authors (M.E., S.N.). Following this, two deductive mappings were carried out by three authors (A.D., M.E., S.N.). In the first mapping, themes were aligned with participant‐defined values, informed by the VSD framework. In the second mapping, themes were analysed according to the Welldoing framework to examine how the co‐design process supported or inhibited participants' satisfaction of needs.

This hybrid analytical approach enables the development of new insights into how co‐design can become more inclusive, ethical, contextual and impactful, particularly for underrepresented groups such as older adults. The analysis enables a deeper understanding about the outcomes of the experience for participants and offers insights about the role of the collaborative practices used in the case.

### 3.5 Ethics

The ethical principles for medical research involving human subjects issued by the World Medical Association Declaration of Helsinki were followed [32]. Written informed consent was obtained before the interviews. All data were handled confidentially, and participants were informed that their participation was entirely voluntary and that they could withdraw from the study at any time without any consequences. The Ethics Review Authority approved the study in Sweden (Dnr 2020–05324).

### 3.6 Case Study COORDINATEs Design Sprint

The prototypes developed through the design sprint are reported elsewhere [33, 34]. However, in the current paper, we present the methods used in undertaking the case study, to enable an exploration of how these methods may be linked with the outcomes of the evaluation.

The case study project used a participatory approach grounded in participatory design theory [35], directly engaging relevant stakeholder groups in the co‐creation of digital prototypes to support housing decisions for older adults. Several months before the initiation of the co‐design activities, a co‐design team was formed, consisting of researchers with expertise in healthcare and architecture, along with representatives from technology and business development. The team engaged in preparatory planning in collaboration with a professional co‐design facilitator experienced in developing digital services for older adults.

This preparatory phase ensured a coherent structure and clear roles for the participants throughout the process.

**Figure 1:**
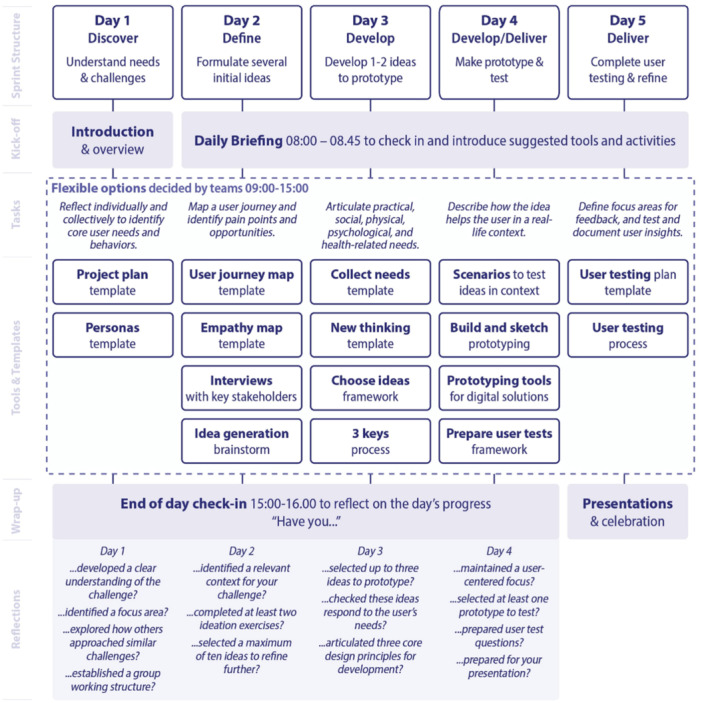
Overview of 5‐day sprint.

A structured 5‐day co‐design sprint [28] was employed based on the Double Diamond Model [36], encompassing the following five iterative phases: Discover (understand needs and challenges), Define (formulate several initial ideas), Develop (develop ideas to prototype), Develop/Deliver (make prototype and test) and Deliver (complete user testing and refine). Full phase descriptions and guiding questions are provided in Figure 1.

Purposive sampling was employed to ensure a diversity of perspectives in the discussions, with the aim of including individuals with relevant knowledge and expertise related to housing and the needs of the aging population. Older adults were included as experts by experience. In this study, older adults refer to individuals aged 65 years or older who live independently in their own homes. The case study project was conducted entirely online, which enabled participation from across Sweden. In total, 18 participants took part: 10 older adults, 4 healthcare professionals and 4 professionals from the fields of technology and architecture (Table 1). Participants were organised into mixed‐composition groups of four to five individuals to capture a range of perspectives. Further detail of the recruitment strategy is reported elsewhere [34].

**Table 1:** Participant characteristics.

Role	Number (*n*)	Gender	Age range/Mean age (years)	Residential location Major City; Town (15k–60k pop); Village (4k–5k pop); Rural
Older adults	10	Women: 6 Men: 4	64–82/74	Town: 7 Village: 1 Rural: 2
Healthcare professionals	4	Women: 3 Men: 1	26–57/39	Town: 1 Rural: 3
Technology and architectural professionals	4	Women: 3 Men: 1	33–55/41	Major city: 3 Town: 1
Total	18	Women: 12 Men: 6	26–82/59	Major city: 3 Town: 9 Village: 1 Rural: 5

The inductive thematic analysis of semi‐structured interview data generated four themes, which are presented below: (1) Respectful dialogues—a bridge to fairness, (2) Autonomy—a source of creativity and frustration, (3) From uncertainty to trust and (4) Feelings of ownership and responsibility. The deductive analysis against the VSD framework shows the process aligned with participant values, and the analysis against the needs framework describes the process as a synergic satisfier.

### 3.7 Respectful Dialogues—a Bridge to Fairness

The participants highlighted the value of working in a group marked by mutual respect, care and attentiveness, an environment that nurtured both protection and affection. Across interviews and observed interactions, the diversity of experiences and skills among older adults and professionals fostered a strong sense of solidarity, where varied perspectives were not only welcomed but actively integrated into the process. There was a strong sense of care in the way the group adapted to one another's needs, particularly for those less familiar with co‐design or digital tools. This consideration fostered a safe, nonjudgmental atmosphere in which participants could engage at their own pace and according to their abilities. The group's inclusive dynamic reflected a genuine effort to protect each other's well‐being, through simple but meaningful actions such as taking regular breaks and offering support when challenges arose.

Several older adults noted that, while much of the content was unfamiliar, they felt proud of what they accomplished. Their self‐esteem was strengthened not only by their individual achievements but also by the collective encouragement and affirmation they received. The process acknowledged that older individuals are often not invited to express their views, and many expressed that this experience gave them a rare opportunity to be heard and respected. Through shared learning, curiosity and empathy, the group created a space where affection was expressed through solidarity, and protection was enacted through sensitivity to one another's circumstances.

### 3.8 Autonomy—a Source of Creativity and Frustration

Overall, the groups functioned independently and autonomously during the sprint. Observations revealed that each group made decisions about the practical implementation on a day‐by‐day basis and had a plan for completing the task, that is, a prototype. Within the groups, routines were established for meeting times, with considerable space for individual needs. In some groups, this was more explicitly defined, while in others, it naturally fell into place how and when the work would be carried out.

Several participants in the groups had commitments they needed to fulfil while the sprint was ongoing, and this applied not only to professionals but also to older adults who had retired. Many participants mentioned that they appreciated the freedom to decide the structure and timing themselves. Working via digital platforms seemed to simplify autonomy and independence, as participants did not need to physically move between locations. Furthermore, digital meetings were seen as beneficial for quickly receiving support from facilitators if the group got stuck. One group expressed that they would have preferred to have access to a digital space they could control independently, rather than being assigned a room they could not manage themselves.

Although the groups, overall, experienced autonomy, challenges emerged. Observations and interviews captured moments of frustration, particularly when participants felt uncertain about expectations or lacked the technical skills to carry out their ideas. In these instances, autonomy became a source of tension, highlighting the delicate balance between freedom and support in collaborative design. In some groups, dynamics emerged where one participant took on a leading role, often driving the work forward and coordinating tasks. When this informal leadership was disrupted due to absence or competing tasks, as evidenced by observational data and interview responses, the group's progress stalled. This imbalance led to frustration, as the responsibility for maintaining momentum was felt to rest on a single individual, without adequate support from the rest of the group.

### 3.9 From Uncertainty to Trust

Most participants expressed considerable uncertainty and a lack of control at the start of the design sprint, as they felt they had neither trust in the process nor confidence in their own role within it. For many, the task felt overwhelming, and it was difficult to understand what the work would ultimately lead to. Older adults described that they were not familiar with the procedure and expressed concerns about not being able to contribute to the group's work. Some mentioned that they felt they needed more knowledge and expertise to complete the task. As the design sprint progressed, participants' perception of trust and control increased, both in relation to the design process and their own ability to contribute to the work. Some aspects highlighted in this context were the importance of good communication within the group, a positive and pleasant atmosphere, and getting to know the other group members. Furthermore, the importance of support from the co‐design team and encouragement to use tools and exercises as part of moving forward in the process emerged.

### 3.10 Feelings of Ownership and Responsibility

The co‐design process was characterised by feelings of ownership and expectations. Across interviews, field notes and observed interactions, many participants expressed a desire to contribute to housing issues affecting older adults, viewing the topic as both urgent and under‐addressed. For older adults, the subject held personal relevance, as they themselves represented the target users of the digital solution. Professionals, on the other hand, described it as an opportunity to influence and shape future practices in their field.

A recurring theme throughout the sprint was a shared sense of responsibility for the outcomes, which often motivated sustained participation and thoughtful contributions to the design work. This was reflected in participants coming up with ideas they wanted to pursue even after the design sprint had concluded. One group, for instance, met with their own initiative after their involvement in the research project to continue the discussion and wrap things up. In another group, a sense of responsibility was expressed in the opposite way. One group participant described that the idea behind their digital solution had potential, however, great dissatisfaction and frustration were expressed in not being able to complete the work to a satisfactory level. Further, our findings revealed a curiosity to learn more about the co‐design method itself, particularly in the form of a design sprint, as well as an interest in gaining insight into conducting such a process entirely digitally.

### 3.11 Evaluating the Experience of Participation Against Participant‐Determined Values

Following the inductive thematic analysis process, transcripts were re‐coded against the values that had been expressed by participants during the workshop process. Table 2 provides examples of participant feedback on the process, aligned with these values.

**Table 2:** Participant descriptions of the process aligning with agreed project values.

Values identified in VSD process	An example of evidence from participants that this was satisfied during the process
Dignity	*We all had different experiences. We were listened to, I thought we listened to everyone, it was very respectful* (Older adult)
Trust	*This was the first time I did something like this so it was really hard to understand what we were going to end up with on Friday, but when I read the information I felt like no, but we will be guided through the process, so maybe I don′t need to know now what will happen on Friday* (Healthcare professional)
Empowerment	*We were actually really impressed that we managed to do this, it was fun because this was very new to us* (Older adult)
Ownership	*You could perhaps see it* [the prototype developed in the group] *as a project together with study associations for adults and also set it up as an education//So, we probably all thought that this was something that we could move forward with* (Older adult)
Usability	*We worked a lot with Miro as a tool, so we tried to follow the structure there. So, it was helpful to go step by step through the program, so to speak. What really helped is that we related to a real event, or experiences of one of the participants in the group* (Technology and architectural professional)
Autonomy	*You got the feeling as time went on that we make this our own, we work the way we want. It felt like a 5‐day sprint, but we are a team and we have a product that we want to make and then we do it this way* (Older adult)

### 3.12 Evaluating the Experience of Participation Against Human Needs Satisfaction

The interpretation of participant feedback according to the Welldoing lens identifies the process as a synergic satisfier, satisfying multiple human needs, from the most basic needs of subsistence and protection to higher‐order needs including identity formation and expressions of freedom. Table 3 summarises where evidence was identified of the satisfaction or violation of each of Max‐Neef's fundamental needs through the processes of participating in the Design Sprint [25].

**Table 3:** Alignment of participant reflections with satisfaction or violation of needs.

Human Need (Max‐Neef, 2007)	Evidence of the process as a satisfier identified	Evidence of the process as a violator identified
Subsistence		✓
Protection	✓	✓
Affection	✓	✓
Understanding	✓	✓
Participation	✓	
Idleness		
Creation	✓	
Identity	✓	
Freedom	✓	

Where participants felt they were free to determine the time they contributed to their groups, this was reflected in positive expressions of satisfying needs for subsistence. For example, IP4 expressed how important it was that people were able to determine the amount of time they could contribute to the project, because of outside commitments:

This thing about working freely during the day and it's up to each person how hard they want to work, and I think it can't be done any other way if you're going to keep going for that long. Because that was my first thought, what kind of people set aside an entire week.

(Older Adult)

On the other hand, where participants felt they needed to prioritise participation over their own needs, this contributed to a negative expression of value in this domain. IP10 for instance, expressed concern that if they didn't compromise other commitments, they felt their group would stop working:

I said I had to drop out for a while because I had to attend a meeting at my job, I can't miss it, I have to jump in. Although I said like this; you can continue without me, but it just didn't work out. So, it was hard with the stress of having to do everything and I didn't have anyone to discuss with, it became frustrating, it was a lot, it was a very emotional week for me.

(Technology and Architectural Professional)

Strong facilitation structures were associated with descriptions of value aligned with the need for protection. IP18 describes a feeling of safety from the provided structure:

There was a model you had to follow. And it's safe, it's good to have something to hold on to. Then you feel like you're moving towards a goal.

(Older Adult)

For IP12, the provision of structured breaks was also recognised as helping others manage energy levels and continue to be able to participate fully:

People who weren't used to [long days], like in my group for example, had to be considered and told that maybe we should take a break and so on.

(Technology and Architectural Professional)

Many participants reported feeling a sense of care and affection develop with others valuing their contributions and perspectives. IP9 described this in the following way:

I have quite a lot to say, and I have some experience and it's great that there's someone who wants to hear it, I thought.

(Older Adult)

Reflections on the sprint describe an appreciation of the opportunity to develop their understanding of others' viewpoints, as well as of the process they were engaged in. While there are multiple descriptions of challenges in understanding an unfamiliar process at the beginning of the process, the structure of the sprint enabled participants to develop more clarity on both the issues and the process as the week progressed. IP15 describes this experience:

You started out pretty blank and then there was a lot of information at the beginning, and there was a lot of new information about how to work so it was a lot at the beginning anyway. And you were pretty tired in the head but then it gradually became clear what you were supposed to do and how you were supposed to work. What we were supposed to do and focus on. So it loosened up more and more.

(Healthcare Professional)

In the participation domain, participants reported value in being involved in shaping agendas and goals, in shared decisions, and in the development of shared ownership. Idleness was not described by participants, although some associated concepts of value were described in the creation domain. IP17 describes excitement at being involved in creating something that they can see tremendous potential in other contexts, while IP15 expressed enjoyment in the stage of creating their idea, including the practical and hands‐on nature of the process:

And then when we got to the actual creation thing, you felt it was full speed, then it became really fun when you started with that instead. To not just try to think, but to do it practically as well.

(Healthcare Professional)

A number of participants described developing their sense of both identity development and expressing freedom and self‐determination in the process. IP8 reflected on their identity as an older person participating in research such as this, noting the importance of recognising their individuality as they age.

I think we often get caught up in thinking that older people need this and that, and then you see older people as a big homogeneous group, which they are not at all. But it's just as different as when we were younger, I mean we have very different needs. But you kind of have a tendency to say; older people have this and that need.

(Older Adult)

IP13 highlighted their group's self‐organisation and delf‐direction, taking ownership of the process and in how they might determine success:

Meeting on Zoom in the group I belonged to once afterwards to kind of talk about how we thought the week was and how it went and so on, we had a bit of an evaluation, you could say. And then it turned out well, then we learned that we could have our own ending in our group. It's really up to us, it doesn't have to be up to you. Because we're adults, so we kind of have to think a little too, right.

(Older Adult)

The results demonstrate links between the way the process was facilitated and both the alignment with the group's prioritised values and the satisfaction of fundamental needs.

**Figure 2:**
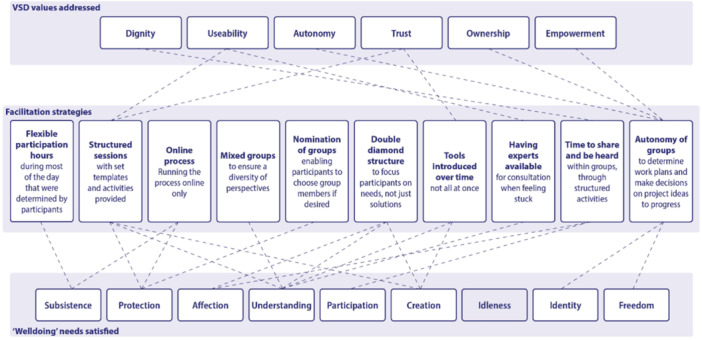
Values.

Figure 2 summarises the identified relationships between key process and facilitation decisions, and the achievement of these outcomes.

In bringing these two approaches together, the Welldoing framework can be seen as describing the impact of participation on individuals, while the connections with values identified through the VSD process provide a framework for process evaluation that is typically used to assess the outcomes from the co‐design project, but in the present study, is used a frame for analysing participant experiences.

## 4 Discussion

This study explored how value emerges through co‐design, using the development of digital housing solutions with a diverse group of stakeholders as a case. We found that the careful facilitation of respectful dialogue was associated with enhanced perceptions of inclusivity and fairness, as well as the formation of meaningful connections among participants.

Group discussions in co‐design are inherently shaped by power dynamics, which are influenced not only by formal roles and affiliations (e.g., whom a participant represents) but also by implicit factors such as age, gender, cultural background and personality traits. The literature acknowledges the variability in how power is managed in participatory processes [37], including challenges specific to work with marginalised communities [38]. However, our findings contribute additional insight into how such dynamics affect individual experiences within a co‐design setting.

In our case, respectful dialogue was largely enabled by the presence of a skilled facilitator who was able to recognise and respond to emerging power imbalances. This facilitation was key in maintaining an environment where all participants, including older adults, felt valued and heard. However, in one instance, the absence of such facilitation led to the breakdown of respectful communication, resulting in participant frustration. Retrospective analysis suggests this frustration was linked to the violation of several psychological needs (as conceptualised in the welldoing model), and a perceived reduction in autonomy (as described in VSD frameworks). This highlights the need for more robust facilitation strategies and ethical sensitivity in future co‐design projects involving diverse and potentially vulnerable groups.

Overall, this study advances our understanding of the mechanisms by which value is generated in co‐design processes. While previous research has examined value perception through quantitative measures [39] and explored participant outcomes qualitatively (e.g. [40]), our work uniquely identifies links between specific facilitation practices and participants' experiences. These findings underscore the importance of trained facilitators in managing power dynamics and promoting equity in collaborative innovation processes, particularly when involving older adults and other underrepresented stakeholders.

In applying these insights into the process of the design sprint, we can make a number of observations of strengths and opportunities to increase the value generated for participants through similar processes. From the analysis, the following can be identified as key strengths of the process in terms of delivering value to participants:

- The careful consideration of group composition, including enabling participants to nominate others' they wished to work with, while also ensuring interdisciplinary teams were formed
- Enabling flexibility of participation, including allowing individuals to choose how much time they commit to the process
- Providing strong structures and processes to ensure teams work at a pace that is appropriate for all
- Including time in the process for people to feel heard: sharing backgrounds, stories and opinions

Taken together, these elements can be seen as positively contributing to the empowerment of individuals who often express feelings of disempowerment [41].

### 4.1 Implications for Policy and Practice

While prior research has primarily examined if and when older adults are involved in the development of digital health interventions [4], the present study extends this understanding by providing nuanced guidance on how to involve older adults and other stakeholders to foster fulfilling, empowering and ethically sound participation. Our findings emphasise the critical role of facilitation strategies that not only enhance inclusivity and fairness but also actively support participants' sense of agency and belonging within co‐design processes. Several practical opportunities to improve future involvement processes emerged from this study:

- Early and clear communication: Providing participants with comprehensive information early in the process about the structure and expectations of activities, such as the design sprint, can reduce uncertainty, promote confidence and enhance engagement.
- Deliberate cultivation of positive team dynamics: Establishing formalised procedures at the outset to nurture constructive collaboration and leadership may be crucial. Examples include organising participants into availability‐based groups and collectively agreeing on ‘ground rules for collaboration' to foster respect, psychological safety and balanced participation.
- Allowing time for ‘productive idleness': Building in deliberate pauses within fast‐paced processes to allow participants time to reflect, digest information and consider alternative perspectives can deepen engagement and creativity. For example, structuring sprints over multiple days with breaks, rather than continuous weekdays, may support thoughtful contributions.

These recommendations must, however, be carefully balanced against the inherent aims of rapid‐cycle innovation approaches such as design sprints. Extending timelines to accommodate ‘productive idleness' risks diluting the process's momentum and potentially creating ‘inhibiting satisfiers', conditions that, while alleviating immediate participant stress, might unintentionally constrain the fulfilment of other critical needs and project objectives.

From a policy perspective, our findings call for health and social care organisations, as well as funders and policymakers, to recognise the complexity of meaningful involvement beyond mere inclusion. Supporting co‐design processes that are ethically robust and participant‐centred may require allocating resources for skilled facilitation, participant preparation and flexible timelines that honour the lived experiences and contributions of older adults and diverse stakeholders. Ultimately, this study contributes to ongoing debates within patient and public involvement literature by foregrounding the delicate balance between process efficiency and participant empowerment. It challenges policymakers and practitioners to critically reflect on how involvement is structured and facilitated, moving beyond box‐ticking approaches towards genuinely equitable and transformative engagement in health innovation.

### 4.2 Limitations

Participants in the present study were recruited because they were able and willing to access digital technologies, potentially over‐emphasising the positive role of online engagement.

After the sprint, individual interviews were conducted via telephone to enhance accessibility and ensure participation regardless of digital literacy or internet access. However, the absence of face‐to‐face interaction may have limited rapport building and the interpretation of non‐verbal cues. The present study is also limited by the evaluation taking place at a project level, rather than a group or individual level. Variations between the approaches taken in each group not able to be linked with observations or outcomes. This also limits to some degree the interpretation of the needs satisfaction because the dataset was analysed as a whole. Much of the focus in the results and discussion is on working with older adults, as these were the largest group, the focus of the case study, and were presumed to be the most vulnerable and least powerful participants. However, the results are described in relation to the experience of all participants to maximise the insight that can be gained across all participant reflections. Future research should continue to build the evidence base for ways of engaging older adults in similar projects so that we can develop a nuanced understanding of how different processes and practices, including VSD approaches, impact their experiences. Such research should document group roles and dynamics to build further knowledge about how older adults establish and carry out their perceived roles in co‐design sprints or other engagement structures. Further longitudinal research with additional groups of older adults would also enable an exploration of the ongoing impact of older adults' participation in the shaping of technologies would help build an understanding of the role of participation in the adoption of technologies, the development of digital literacy, and of digital competency in this group.

## 5 Conclusion

This study demonstrates that involving older adults in the co‐design of digital products and services does more than shape usable tools: it creates opportunities to generate value for participants themselves. By applying a hybrid thematic analysis, informed by both VSD and the Welldoing framework, we have shown how facilitation decisions and process structures can enable or inhibit the satisfaction of participants' values and needs. Respectful dialogue, trust‐building, autonomy and ownership emerged not simply as desirable process features but as essential conditions for fostering dignity, inclusion and well‐being during co‐design. These findings underscore the importance of viewing co‐design not as a neutral method, but as a practice that actively produces social, ethical and emotional outcomes. For practitioners and researchers, this highlights the need to invest in facilitation strategies that balance freedom with support, create space for diverse voices and establish conditions where participants feel both safe and empowered. Co‐design processes with older adults should respect heterogeneity, support agency and promote authentic collaboration. Paying careful attention to the alignment between facilitation, values and human needs provides the opportunity to not only enhance the quality and relevance of digital innovations, but also to ensure that the participation itself is a meaningful, empowering and well‐being‐enhancing experience.

## Author Contributions

**Susanna Nordin:** data curation, formal analysis, investigation, methodology, validation, project administration, writing – original draft. **Aaron Davis:** methodology, formal analysis, visualisation, writing – review and editing, **France Légaré:** conceptualisation, funding acquisition, methodology, writing – review and editing. **Allyson Jones:** conceptualisation, methodology, validation, writing – review and editing. **Louise Meijering:** conceptualisation, methodology, validation, writing – review and editing. **Marie Elf:** conceptualisation, formal analysis, funding acquisition, methodology, project administration, validation, writing – review and editing, supervision.

## Ethics Statement

The Ethics Review Authority approved the study in Sweden (Dnr 2020–05324).

## Consent

Written informed consent was obtained from the participants.

## Take‐Home Message

Thoughtful facilitation in co‐design can transform participation into a source of empowerment and well‐being, ensuring that older adults' voices shape both the process and the product.

## Conflicts of Interest

The authors declare no conflicts of interest.

## Supporting information

Supporting materials for manuscript.

## Data Availability

The data that support the study findings are available from the corresponding author upon reasonable request. Participant data will remain confidential.
