# Supplementary material for: Designing Meaningful Engagement for Older Adults: An Evaluation of Participation in a Five‐Day Co‐Design Sprint
Source: Health Expect. 2026 Jan 12;29(1):e70555. doi: 10.1111/hex.70555 (PMC12793890; doi:10.1111/hex.70555)
Supplement: Supplementary file 1 — Supporting materials for manuscript. [file HEX-29-e70555-s001.docx]

Supplementary materials 1.

**Interview guide**

**Main questions**

- What motivated you to participate in this workshop, which was structured as a five-day design sprint?
- **Could you provide an overview of the week’s activities, from Monday to Friday?**
- How did you experience the sprint? What worked well, and what worked less well?
- What was your impression of the collaboration? (e.g., group dynamics, your role within the group)
- What factors facilitated or hindered the expression of different perspectives? (e.g., thoughts, ideas, values, experiences)
- The groups included participants with diverse backgrounds, how do you view that?
- Approximately how much time did your group spend on this work? Was the time sufficient?
- Regarding the digital solution your group developed, how could it be made accessible to older adults?

**Follow-up and closing questions**

- In what ways did this workshop differ from others you have participated in?
- Can you see yourself participating in similar workshops in the future? Why or why not?
- Is there anything else you would like to add that we haven't touched on?
